# Ecology Drives the Global Distribution of Human Diseases

**DOI:** 10.1371/journal.pbio.0020186

**Published:** 2004-06-15

It's no surprise that the Amazonian rainforest contains far more species than, say, the Siberian tundra. Over 50% of the world's species live in tropical rainforests, which cover just 6% to 7% of the earth's terrestrial surface. That the number of marine and terrestrial species declines with distance from the equator is a well-documented phenomenon called the latitudinal species diversity gradient. What's proven challenging, however, is figuring out what drives this pattern. Over 30 hypotheses have been proposed in the past two decades, but only four have garnered serious attention. These four focus on variables relating to area and energy factors, geographic constraints, and habitat diversity. Understanding the factors—both contemporary and ancient—responsible for the diversity gradient could help answer one of the fundamental questions in evolutionary ecology: what regulates species diversity? But teasing out the likely mechanisms behind this diversity has practical implications as well: mounting evidence suggests that ecological and climatic conditions influence the emergence, spread, and recurrence of infectious diseases. Global climate change is likely to aggravate climate-sensitive diseases in unpredictable ways.[Fig pbio-0020186-g001]

**Figure pbio-0020186-g001:**
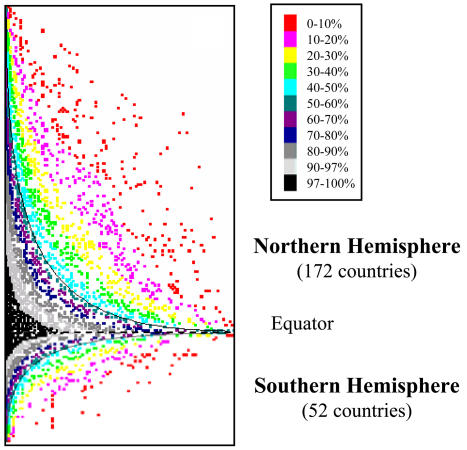
The number of pathogen species increases towards the equator

Increasingly, public health programs aimed at preventing and controlling disease outbreaks are considering aspects of the ecology of infectious diseases—how hosts, vectors, and parasites interact with each other and their environment. The hope is that by understanding how ecological factors impact the global distribution of parasitic and infectious diseases, public health officials can predict and contain future outbreaks. Even though parasitic and infectious organisms account for a major fraction of the biological diversity on the planet, few studies have analyzed the factors affecting the spatial distribution of these organisms or attempted to quantify their contribution to biodiversity. In this issue, Vanina Guernier, Michael Hochberg, and Jean-François Guégan address the influence of ecological factors on the biological diversity and distribution of parasitic and infectious diseases and find that climatic factors are the most important determinant of the global distribution of human pathogens.

The current understanding of human disease and availability of complete datasets on many parasitic and infectious diseases, the researchers explain, present a unique opportunity to explore the relationship between parasitic and infectious disease species richness (defined in their study as total number of pathogens within a given country's borders) and latitude. This information, in turn, can help identify potential factors that affect diversity gradients. After compiling epidemiological data on 332 different human pathogens across 224 countries, Guernier et al. used sophisticated statistical modeling methods to identify and characterize the influence of a number of potential contributing factors on species richness. After adjusting the model to control for cofactors that might influence the relationship between latitude and species richness indirectly rather than directly (cofactors such as the size of countries and demographic, economic, and environmental variables), the researchers confirmed that, on average (seven times out of ten), tropical areas harbor a larger number of pathogen species than more temperate areas. In other words, the species richness of human pathogens follows the same pattern seen in other species.

These results, Guernier et al. argue, suggest that the latitudinal species diversity gradient “might be generated in large part by biotic interactions.” This in turn indicates that current estimates of species diversity, which ignore parasites and infectious organisms, are “substantially underestimated.” The authors went on to explore groupings of individual pathogen species within larger parasitic and infectious disease communities along the gradient and found that species present at northern latitudes are a subset of those present in equatorial areas, rather than a different set of species (a phenomenon called “nestedness”). Since nestedness is strongly associated with latitude, which is typically used as a proxy for a range of climatic factors, the researchers investigated the relationship between various climatic variables and pathogen diversity. The climatic variable most strongly correlated with diversity was the maximum range of precipitation of a region.

The finding that climatic factors are largely responsible for the spatial distribution of human pathogens has important implications for predicting and managing future infectious disease outbreaks. These results counter the conventional assumption that socioeconomic conditions are the most important factor in controlling disease, indicating that global climate change could have far more significant effects on global patterns of disease, with diseases once relegated to the tropics migrating to temperate zones, for example. Identifying the links between ecology and disease, however, could lay the foundation for effective preventive strategies.

